# Acidosis-Induced Dysfunction of Cortical GABAergic Neurons through Astrocyte-Related Excitotoxicity

**DOI:** 10.1371/journal.pone.0140324

**Published:** 2015-10-16

**Authors:** Li Huang, Shidi Zhao, Wei Lu, Sudong Guan, Yan Zhu, Jin-Hui Wang

**Affiliations:** 1 Department of Pathophysiology, Bengbu Medical College, Bengbu Anhui, China 233000; 2 Institute of Biophysics, Chinese Academy of Sciences, Beijing China 100101; 3 Collaborative Innovation Center for Neurodegenerative Disorders in Shandong, Qingdao University, Medical College, 38 Dengzhou, Shandong China 266021; University of North Texas Health Science Center, UNITED STATES

## Abstract

**Background:**

Acidosis impairs cognitions and behaviors presumably by acidification-induced changes in neuronal metabolism. Cortical GABAergic neurons are vulnerable to pathological factors and their injury leads to brain dysfunction. How acidosis induces GABAergic neuron injury remains elusive. As the glia cells and neurons interact each other, we intend to examine the role of the astrocytes in acidosis-induced GABAergic neuron injury.

**Results:**

Experiments were done at GABAergic cells and astrocytes in mouse cortical slices. To identify astrocytic involvement in acidosis-induced impairment, we induced the acidification in single GABAergic neuron by infusing proton intracellularly or in both neurons and astrocytes by using proton extracellularly. Compared the effects of intracellular acidification and extracellular acidification on GABAergic neurons, we found that their active intrinsic properties and synaptic outputs appeared more severely impaired in extracellular acidosis than intracellular acidosis. Meanwhile, extracellular acidosis deteriorated glutamate transporter currents on the astrocytes and upregulated excitatory synaptic transmission on the GABAergic neurons. Moreover, the antagonists of glutamate NMDA-/AMPA-receptors partially reverse extracellular acidosis-induced injury in the GABAergic neurons.

**Conclusion:**

Our studies suggest that acidosis leads to the dysfunction of cortical GABAergic neurons by astrocyte-mediated excitotoxicity, in addition to their metabolic changes as indicated previously.

## Introduction

Acidosis, a base-acid imbalance, is associated with many diseases in the clinic practices including diabetes mellitus as well as the disorders in the urinary, respiratory and gastrointestinal systems [1–4]. The patients suffered from acidosis often express cognitive deficits, such as anxiety, convulsion and unconsciousness [2,3,5], which result from acidosis-induced neuron dysfunction. The elucidation of their mechanism is critical for exploring new approach that protects brain function from acidosis. Previous studies focused on elucidating a role of metabolic alternations in neuron dysfunction, including intracellular enzyme activities [6], proton accumulation [7,8], acid-sensing ion channels [9–14], voltage-gated sodium channels [15] and voltage-gated calcium channels [16]. How these mechanisms occur in the specific types of the neurons and glia cells for acidosis-induced neuropathy is largely unknown.

The brain functions are maintained by the physiological interaction and balance between cerebral excitatory and inhibitory neurons, in which the GABAergic neurons coordinate the activities of principal neurons for the brain to program neuronal codes and to manage well-organized cognitions [17–20]. It is well known that the GABAergic neurons are vulnerable to pathological factors [21–23], and acidosis leads to brain dysfunction by impairing the GABAergic neurons [15,24]. As intracellular metabolic alternations for acidosis-induced neuron injury are well studied, we aim to examine a role of their neighboring cells in the acidosis-induced injury of GABAergic neurons. An interaction between neurons and glia cells fulfills the brain functions [25,26], and the molecules related to neuron-astrocyte interaction affect the brain functions [27,28]. Here, we focus on investigating how the astrocytes lead to GABAergic neuron injury during acidosis.

To study a role of astrocyte-related excitotoxicity in neuron injury, our strategies are given below. We induced acidosis in single neuron by infusing protons intracellularly or in both neurons and astrocytes by washing-on protons extracellularly. The severer effects of extracellular acidification than intracellular acidification on the neurons indicated the involvement of the astrocytes in acidosis-induced neuron injury. This indication would be granted if the astrocytes’ function decreased during acidification. If the function of glutamate transporter on the astrocytes decreased and the activity of excitatory synapses on the neurons increased during acidosis, astrocyte-related excitotoxicity would presumably be involved in neuron injury. This indication would be granted if the effect of extracellular acidosis on neurons was partially reversed by inhibiting their glutamate receptors, i.e., both cellular metabolic deficit and astrocyte-related glutamate toxicity are involved in acidosis-induced neuron impairment.

## Results

To separate the roles of astrocyte-related excitotoxicity and metabolic deficits in acidosis-induced neuron injury, we induced acidosis in a GABAergic neuron by infusing protons intracellularly or in both neurons and glia cells by washing protons extracellularly. The severer effects of extracellular acidification than intracellular acidification on GABAergic cells indicated the involvement of neuron-glia interaction in acidosis-induced GABAergic neuron injury. If glutamate transporters on the astrocytes decreased and excitatory synaptic transmission on the GABAergic neurons increased in acidosis, the role of astrocyte-related excitotoxicity in acidosis-induced GABAergic neuron injury would be indicated. This indication would be strengthened if the effect of extracellular acidification on the GABAergic neurons is partially reversed by blocking their glutamate receptors.

### Intracellular and extracellular acidosis impairs cortical GABAergic neurons differently

The functions of GABAergic neurons in cortical slices were evaluated by analyzing their intrinsic properties and synaptic outputs before and after intracellular or extracellular acidifications. Spontaneous inhibitory postsynaptic currents (sIPSC) were recorded on pyramidal neurons to evaluate their receptions to synaptic outputs of GABAergic neurons. Sequential action potentials, refractory periods and threshold potentials were recorded on the GABAergic neurons to assess their active intrinsic properties.

**Fig 1:**
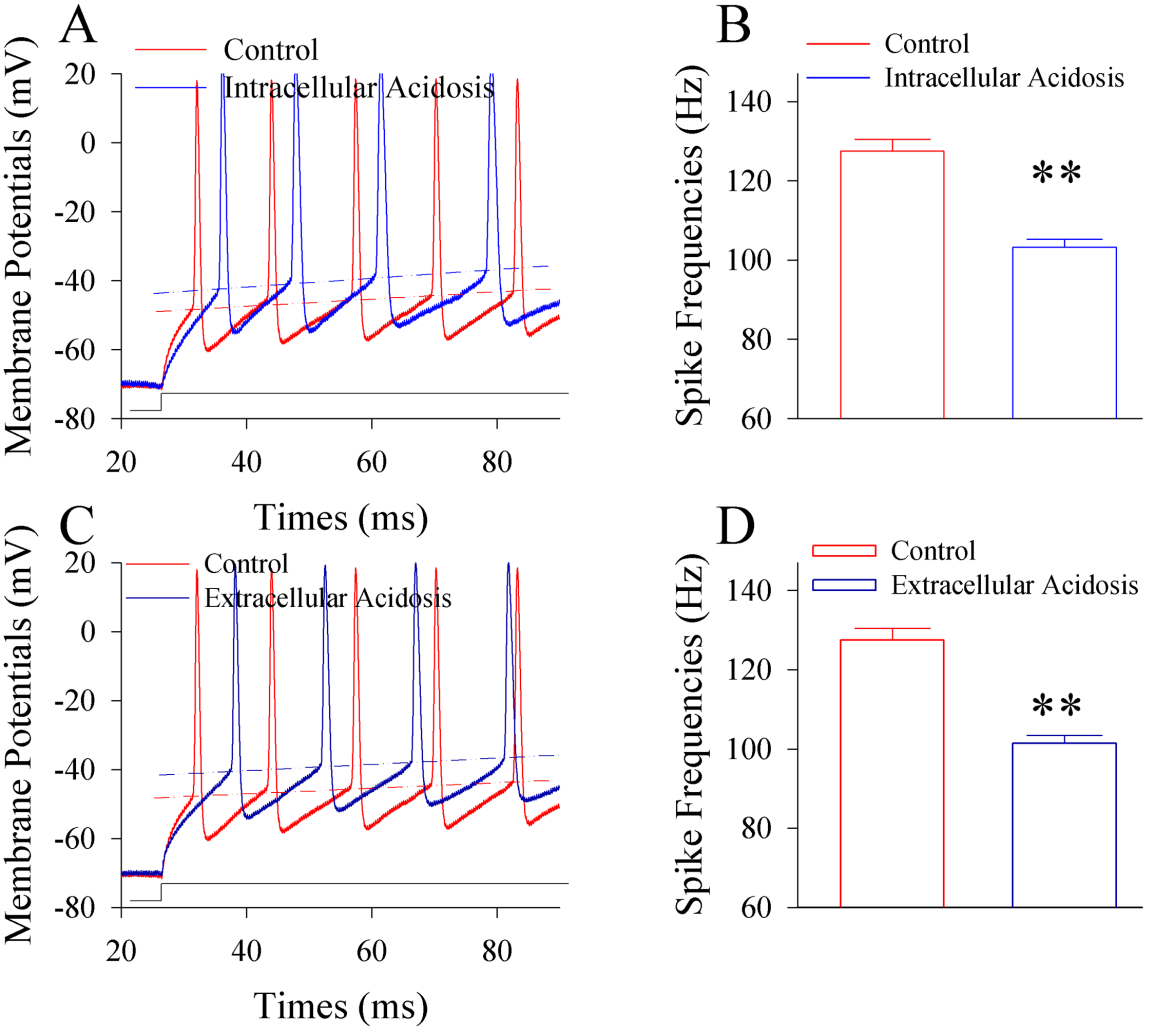
Extracellular and intracellular acidosis impairs the production of action potentials at the cortical GABAergic neurons. Sequential spikes of GABAergic neurons in cortical slices were evoked by injecting depolarization pulse (200 ms) and recorded by using whole-cell current-clamp. Extracellular acidosis was made by perfusing the cortical slices with the acidic ACSF (pH 6.75) after the control ACSF (pH 7.35). Intracellular acidosis was made by using the recording pipettes whose tips were filled with control pipette solution (pH 7.35) and back-filled with acidification pipette solution (pH 6.75). **A)** shows the evoked spikes under the control (red trace) and subsequent intracellular acidification (blue trace), respectively. **B)** shows the values of spike frequencies under the conditions of control (pH 7.35, red bar) and intracellular acidification (pH 6.75; blue bar). Two asterisks show p<0.01 (n = 15, paired t-test). **C)** shows the spikes under the control (red trace) and extracellular acidification (dark blue), respectively. **D)** shows the values of spike frequencies under the conditions of control (pH 7.35, red bar) and extracellular acidification (pH 6.75; dark-blue). Two asterisks show p<0.01 (n = 15, paired t-test). Dash-lines in **A)** & **C)** show the levels of threshold potentials.

**Fig 2:**
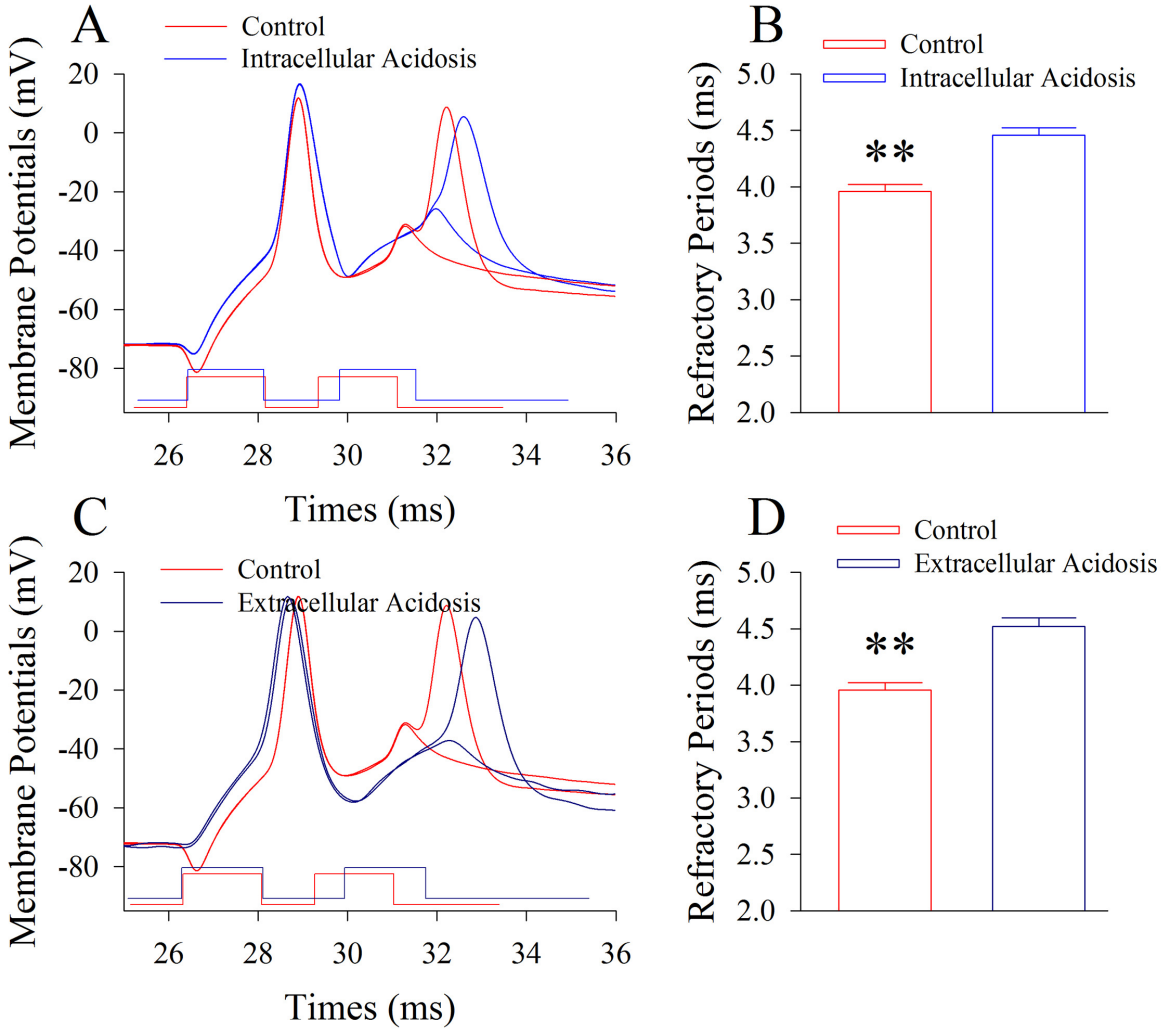
Extracellular and intracellular acidosis prolongs the refractory periods of action potentials at the cortical GABAergic neurons. Refractory periods were measured by injecting paired-depolarization pulses and recorded under whole-cell current-clamp. **A)** shows the measurements of refractory periods under the control (red trace) and subsequent intracellular acidification (blue), respectively. **B)** shows the averaged values of spike refractory periods under the conditions of control (pH 7.35, red bar) and intracellular acidification (pH 6.75; blue). Two asterisks show p<0.01 (n = 15, paired t-test). **C)** shows the measurement refractory periods under the control (red trace) and extracellular acidification (dark blue), respectively. **D)** shows the values of refractory periods under the conditions of control (pH 7.35, red bar) and extracellular acidification (pH 6.75; dark-blue). Two asterisks show p<0.01 (n = 15, paired t-test).

The influences of intracellular and extracellular acidosis on the spiking ability and active intrinsic properties of cortical GABAergic neurons are shown in Fig 1. Intracellular acidosis appears to reduce the spiking ability of GABAergic neurons (Fig 1A). Spike frequencies at the maximal level of input-outputs are 127.5±2.99 Hz before acidosis and 103.3±1.97 Hz after acidosis (Fig 1B; p<0.01, n = 15; paired t-test). Extracellular acidosis appears also to attenuate the spiking ability of GABAergic neurons (Fig 1C). Spike frequencies at the maximal level of input-output are 127.5±2.99 Hz before acidosis and 100.4±2.0 Hz after acidosis (Fig 1D; p<0.01, n = 15; paired t-test). Thus, both intracellular and extracellular acidifications impair GABAergic neurons in their spiking ability. In terms of active intrinsic property, both intracellular and extracellular acidifications elevate threshold potentials to fire spikes (dash lines in Fig 1A and 1C) and prolong spike refractory periods (Fig 2A and 2C). The refractory periods are 3.96±0.06 ms before intracellular acidosis and 4.45±0.06 ms after acidosis (Fig 2B; p<0.01, n = 15; paired t-test). The refractory periods are 3.96±0.06 ms before extracellular acidosis and 4.53±0.07 ms after acidosis (Fig 2D; p<0.01, n = 15; paired t-test). The results indicate that both intracellular acidosis and extracellular acidosis impair GABAergic neurons in their active intrinsic properties to lower spiking capability.

**Fig 3:**
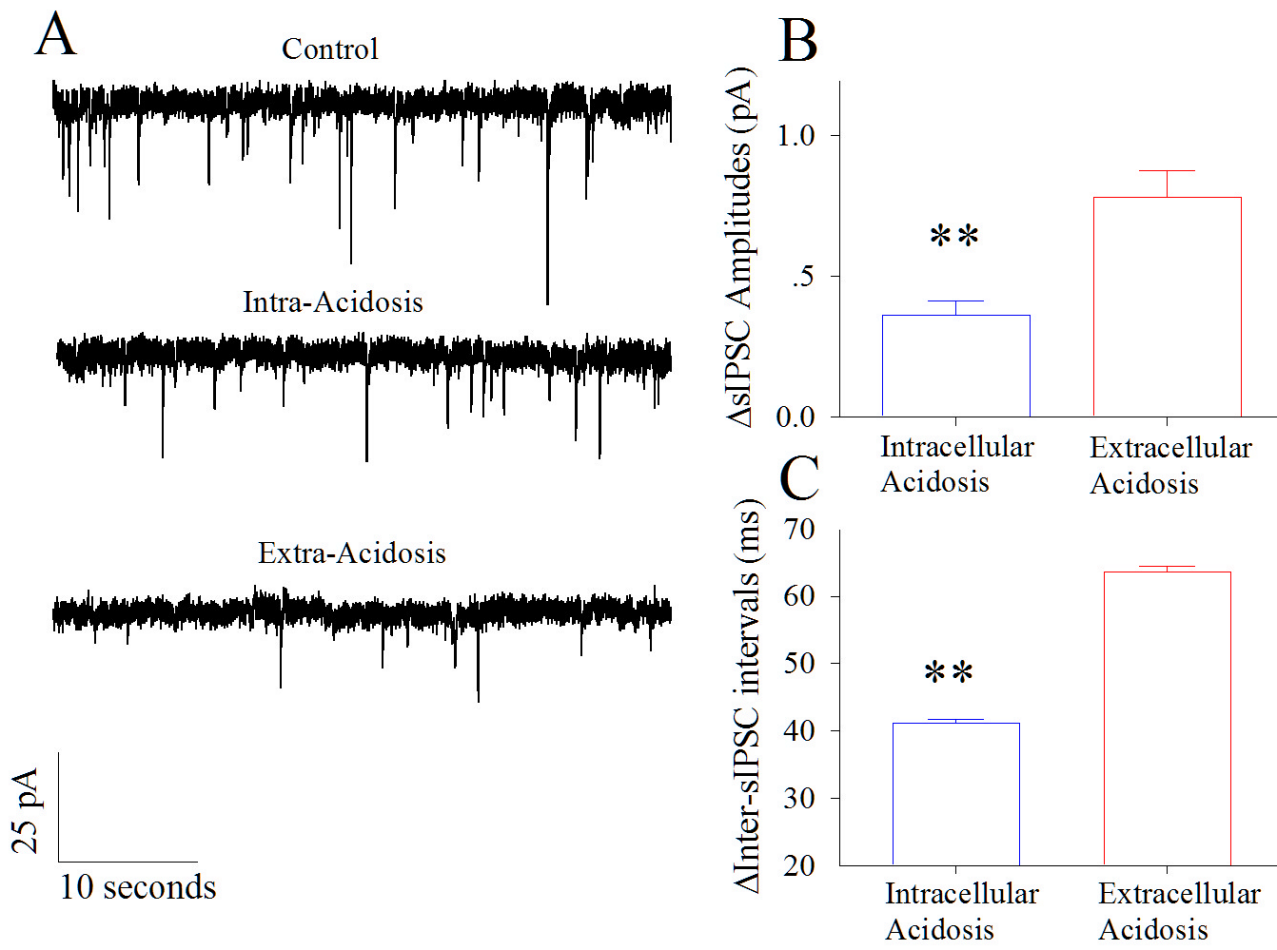
Extracellular acidosis impairs GABAergic synaptic transmission at cortical pyramidal neurons dominantly. Spontaneous IPSCs (sIPSC) were recorded by whole-cell voltage-clamp without stimulating presynaptic axons under the conditions of control and then extracellular acidification versus of control and intracellular acidification. **A)** shows the recorded sIPSCs under the control (top trace), intracellular acidification (middle trace) and extracellular acidification (bottom trace). **B)** illustrates the differences of sIPSC amplitudes between control and intracellular acidosis (∆IPSC amplitudes, red bar) as well as the ∆IPSC amplitudes between control and extracellular acidosis (blue bar; two asterisks, p<0.01, n = 15; one-way ANOVA). **C)** illustrates the differences of inter-sIPSC intervals between control and intracellular acidosis (∆inter-IPSC interval, red bar) as well as ∆inter-IPSC intervals between control and extracellular acidosis (blue bar; two asterisks, p<0.01, n = 15; one-way ANOVA).

The influences of intracellular and extracellular acidosis on the synaptic outputs of GABAergic neurons are shown in Fig 3. Intracellular acidosis appears to reduce GABAergic synaptic transmission (Fig 3A). The difference of sIPSC amplitudes (∆sIPSC amplitudes) between control and intracellular acidosis is 0.38±0.03 pA. ∆sIPSC amplitude between control and extracellular acidosis is 0.77±0.08 pA. The net decreases in sIPSC amplitudes by extracellular acidosis versus intracellular acidosis are statistical difference (p<0.01, n = 15; one-way ANOVA in Fig 3B). Furthermore, the difference of inter-sIPSC intervals (i.e., ∆inter-sIPSC intervals) before and after intracellular acidosis is 41.5±1.5 ms. ∆inter-sIPSC interval before and after extracellular acidosis is 63.7±2.1 ms. The net decreases in sIPSC frequencies by extracellular acidosis vs. intracellular acidosis are statistical difference (p<0.01, n = 15; one-way ANOVA in Fig 3C). These results indicate that both intracellular acidosis and extracellular acidosis deteriorate GABAergic synaptic functions, but extracellular acidosis more severely impairs the synaptic outputs from GABAergic neurons.

**Fig 4:**
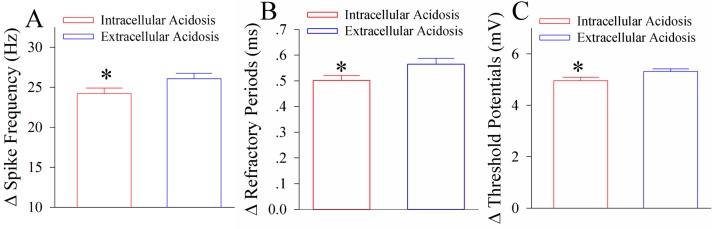
Extracellular acidosis impairs the active intrinsic properties of the cortical GABAergic neurons dominantly. **A)** shows the differences of spike frequencies between control and intracellular acidosis (red bar) as well as between control and extracellular acidosis (blue bar; asterisk, p<0.05; one-way ANOVA). **B)** shows the differences of spike refractory periods between control and intracellular acidosis (red bar) as well as between control and extracellular acidosis (blue bar; one asterisk, p<0.05; one-way ANOVA). **C)** shows the differences of spike threshold potentials between control and intracellular acidosis (red bar) as well as between control and extracellular acidosis (blue bar; one asterisk, p<0.05; one-way ANOVA).

In addition, the influences of extracellular acidosis on spiking abilities and intrinsic properties at GABAergic neurons are significantly severer than the influences of intracellular acidosis (Fig 4A–4C). Therefore, the impairment of GABAergic neurons in their excitability and synaptic outputs is more severe in extracellular acidosis than intracellular acidosis. In terms of the mechanisms underlying these facts, we hypothesized that extracellular acidosis may influence the nerve cells around GABAergic neurons, such as the astrocytes and the glutamatergic neurons, to indirectly make additional impairment to GABAergic neurons, besides the effect of intracellular protons on GABAergic neurons. This hypothesis was based on the reports that the astrocytes were involved in neuronal injury [29,30] and the impairment of GABAergic neurons was induced by neuronal excitotoxicity [22,31,32].

### Extracellular acidosis impairs glutamate transporters on cortical astrocytes

**Fig 5:**
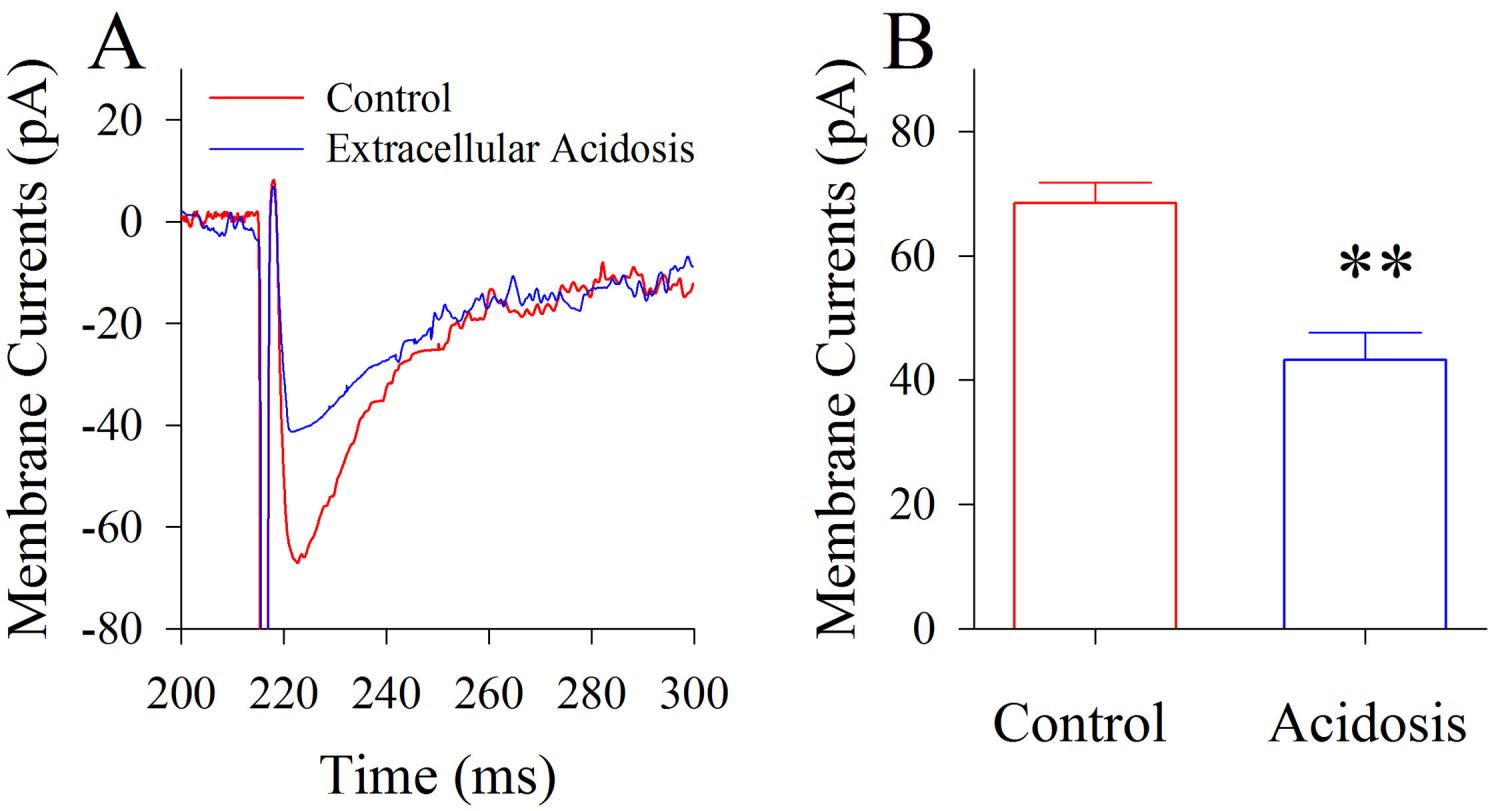
Extracellular acidosis impairs the glutamate transporter current (GTC) on the cortical astrocytes. The GTCs were recorded on cortical astrocytes and evoked by stimulating presynaptic axons. **A)** shows the superimposed waveforms of GTCs before (red trace) and after extracellular acidification (blue trace). **B)** shows the averaged values of GTCs before (red bar) and after extracellular acidification (blue bar; two asterisks, p<0.01, n = 16; paired t-test).

Glutamate transporter currents (GTC) were recorded at the astrocytes in cortical slices to assess astrocytic function since the glutamate uptake from synaptic clefts was one of major astrocytic functions [33–35]. Astrocytic GTCs were recorded by whole-cell voltage-clamp when the presynaptic axons were stimulated. Extracellular acidosis appears to reduce astrocytic GTCs (Fig 5A). The averaged GTC amplitudes are 68.48±3.28 pA under the control and 43.2±4.38 pA after acidosis (asterisks, p<0.01, n = 16; paired t-test in Fig 5B). It is noteworthy that the recorded GTCs are blocked by 10 μM TBOA (glutamate transporter antagonist; [36,37], i.e., the activity of glutamate transporters is recorded (S1 Fig). This result indicates that extracellular acidosis impairs the function of astrocytic glutamate transporter and the uptake of glutamates, such that the accumulated glutamates in synaptic clefts make GABAergic neurons injured.

To examine this indication, we recorded spontaneous excitatory postsynaptic currents (sEPSC) on GABAergic neurons to analyze whether extracellular acidosis enhanced glutamatergic synapses on these neurons. In addition, we recorded the GABAergic neuron activities in the presence of glutamate receptor antagonists to analyze whether the inhibition of glutamatergic synapses could reverse GABAergic neuron injury induced by extracellular acidosis.

### Extracellular acidosis strengthens glutamatergic synaptic transmission on GABAergic neurons

**Fig 6:**
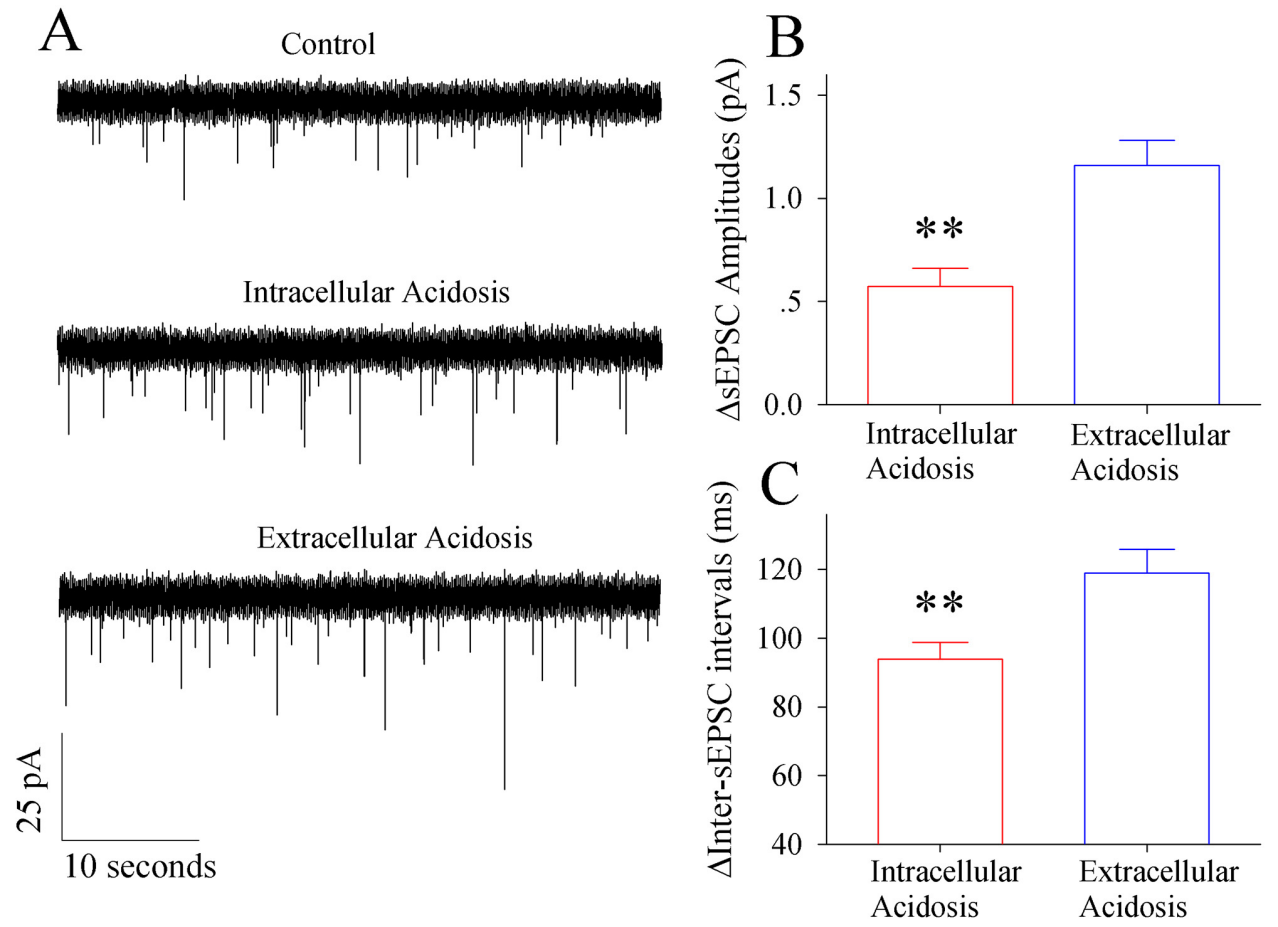
Extracellular acidosis upregulates glutamatergic synaptic transmission at cortical GABAergic neurons dominantly. Spontaneous EPSCs were recorded on GABAergic neurons by whole-cell voltage-clamp without stimulating presynaptic axons. **A)** shows the recorded sEPSCs under the control (top trace), intracellular acidification (middle trace) and extracellular acidification (bottom trace). **B)** illustrates the differences of sEPSC amplitudes between intracellular acidosis and control (∆EPSC amplitudes, red bar) as well as the differences between extracellular acidosis and control (∆EPSC amplitudes, blue bar; p<0.01, n = 15; one-way ANOVA). **C)** shows the differences of inter-sEPSC interval between intracellular acidosis and control (∆inter-IPSC interval, red bar) as well as the differences between extracellular acidosis and control (blue bar; p<0.01, n = 15; one-way ANOVA).

The effects of extracellular acidosis or intracellular acidosis on the glutamatergic synapses at the GABAergic neurons are showed in Fig 6. Based on the recorded sEPSCs, extracellular or intracellular acidosis appears to increase the activity of excitatory synapses on GABAergic neurons (Fig 6A). The difference of sEPSC amplitudes (∆sEPSC amplitudes) before and after intracellular acidosis is 0.55±0.05 pA. ∆sEPSC amplitude before and after extracellular acidosis is 1.17±0.12 pA. The net changes in sEPSC amplitudes by extracellular acidosis and intracellular acidosis are statistic difference (p<0.01, n = 15, one-way ANOVA; Fig 6B). Furthermore, the difference of inter-sEPSC intervals (∆inter-sEPSC intervals) before and after intracellular acidosis is 93.5±5.1 ms. ∆inter-sEPSC interval before and after extracellular acidosis is 117.9±7 ms. The net increases in sEPSC frequencies by extracellular acidosis and intracellular acidosis are statistic difference (p<0.01, n = 15; one-way ANOVA; Fig 6C). These results indicate that extracellular acidosis dominantly strengthens presynaptic glutamate release and postsynaptic glutamate receptor activity, such that glutamatergic synaptic transmission on the GABAergic neurons is upregulated. This change cannot be corrected during extracellular acidosis as the astrocytic glutamate transporters are dysfunctional to re-uptake the released glutamates (Fig 5).

The increase of presynaptic glutamate release and the decrease of astrocytic activities lead to the glutamate accumulation in synaptic cleft, so that these glutamates make excitotoxicity on the GABAergic neurons, i.e., extracellular acidosis dominantly impairs GABAergic neurons. If it is a case, the inhibition of glutamatergic receptor on the GABAergic neurons should partially reverse their injury by extracellular acidosis.

### Antagonists of glutamatergic receptors reverse GABAergic impairment by extracellular acidosis

**Fig 7:**
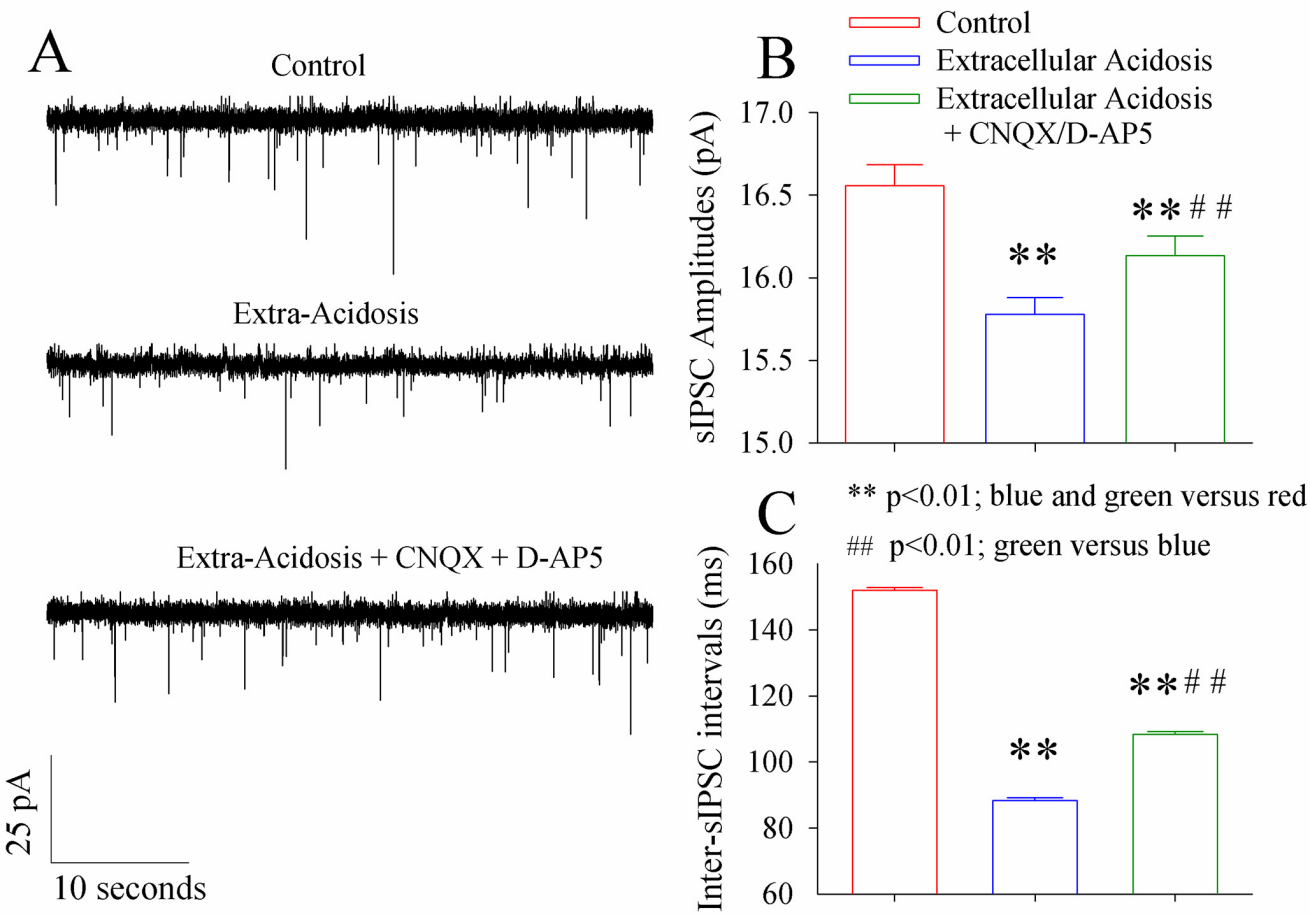
The inhibition of glutamate receptors partially reverses the impairment of GABAergic synaptic outputs to cortical pyramidal neurons induced by extracellular acidosis. sIPSCs were recorded by whole-cell voltage-clamp under the conditions of sequential manipulations, i.e., control, extracellular acidosis and extracellular acidosis plus 10 μM CNQX and 40 μM D-AP5. **A)** shows the recorded sIPSCs under the control (top trace), extracellular acidification (middle trace) and extracellular acidification plus glutamate receptor blockers (bottom trace). **B)** shows the averaged values of sIPSC amplitudes under the conditions of control (red bar), extracellular acidosis (blue bar) and extracellular acidosis plus glutamate receptor blockers (green bar; two asterisks, p<0.01; ##, p<0.01; n = 15; one-way ANOVA). **C)** shows the averaged values of inter-sIPSC intervals under the conditions of control (red bar), extracellular acidosis (blue bar) and extracellular acidosis plus glutamate receptor blockers (green bar; two asterisks, p<0.01; ##, p<0.01; n = 15; one-way ANOVA).

The influence of inhibiting glutamate receptors on the synaptic outputs from GABAergic neurons is showed in Fig 7. The dysfunction of GABAergic synapses induced by extracellular acidosis appears to be partially reversed by 10 µM CNQX/40 µM D-AP5 (Fig 7A). sIPSC amplitudes are 16.52±0.17 pA under the control, 15.76±0.15 pA during extracellular acidosis and 16.1±0.16 pA during extracellular acidosis in the presence of CNQX and D-AP5 (Fig 7B; p<0.01, n = 12, one-way ANOVA). Inter-sIPSC intervals are 151.5±1.1 ms under control, 87.6±1.5 ms in extracellular acidosis and 106.1±2.1 ms during extracellular acidosis plus CNQX/D-AP5 (p<0.01, n = 12, one-way ANOVA in Fig 7C). This partial reverse of acidosis-induced GABAergic synapse injury by blocking glutamate receptors indicates that the elevated glutamates during extracellular acidosis impair GABAergic neurons through excitotoxicity.

**Fig 8:**
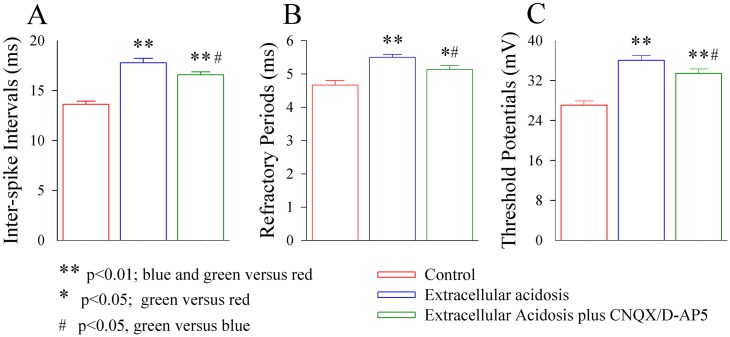
The inhibition of glutamate receptors partially reverses the impairment of intrinsic properties at cortical GABAergic neurons induced by extracellular acidosis. Sequential spikes, threshold potentials and refractory period at GABAergic neurons were recorded by whole-cell voltage-clamp under the conditions of sequential manipulations, i.e., control, extracellular acidosis and extracellular acidosis plus 40 μM D-AP5 and 10 μM CNQX. **A)** shows the averaged values of spike frequency under the conditions of control (red bar), extracellular acidosis (blue bar) and extracellular acidosis plus glutamate receptor blockers (green bar). **B)** shows the averaged values of spike refractory periods under the conditions of control (red bar), extracellular acidosis (blue bar) and extracellular acidosis plus glutamate receptor blockers (green bar). **C)** shows the averaged values of spike threshold potentials under the conditions of control (red bar), extracellular acidosis (blue bar) and extracellular acidosis plus glutamate receptor blockers (green bar). Two asterisks present p<0.01, such as blue and green bars versus red bar. An asterisk presents p<0.05, such as green bar versus red bar. # presents p<0.05, such as green bar versus blue bar (n = 15; one-way ANOVA).

We also examined the influences of inhibiting glutamatergic receptors on the intrinsic properties of GABAergic neurons (Fig 8). Extracellular acidosis-induced impairments of their intrinsic properties appear to be partially reversed by 10 μM CNQX and 40 μM D-AP5. Inter-spike intervals are 13.6±0.3 ms under the control, 17.8±0.4 ms in extracellular acidosis and 16.5±0.3 ms during extracellular acidosis in the presence of CNQX and D-AP5 (Fig 8A; p<0.01, n = 12, one-way ANOVA). The refractory periods are 4.7±0.13 ms under the control, 5.5±0.1 ms during extracellular acidosis and 5.1±0.12 ms during extracellular acidosis plus CNQX/D-AP5 (Fig 8B; p<0.01, n = 12, one-way ANOVA). The threshold potentials are 27.1±0.81 mV under control, 36.1±0.9 mV during extracellular acidosis and 33.4±0.89 ms during extracellular acidosis plus CNQX/D-AP5 (Fig 8C; p<0.01, n = 12, one-way ANOVA). These data strengthen an indication that the elevated glutamates during extracellular acidosis impair GABAergic neurons through excitotoxicity.

## Discussion

**Fig 9:**
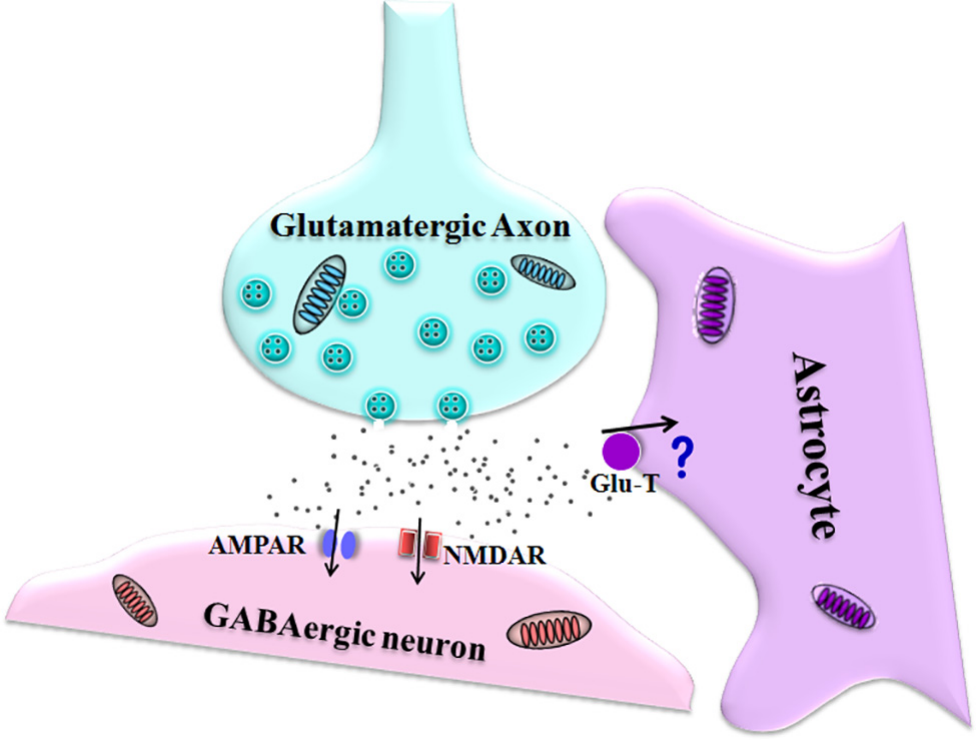
The dysfunction of glutamate transporter in the astrocyte leads to the impairment of GABAergic neuron during acidosis. Extracellular acidification impairs the function of astrocytic glutamate transporter (Glu-T), and the subsequent glutamate accumulation deteriorates GABAergic neurons through activating ionotropic glutamate receptors, such as NMDAR and AMPAR.

We examined the involvement of cortical astrocytes in the acidosis-induced injury of GABAergic neurons. Extracellular acidosis impairs the GABAergic neurons in active intrinsic properties and synaptic outputs more severely, in comparison with intracellular acidosis (Figs 1–4). Extracellular acidosis also impairs cortical astrocytes in terms of the re-uptake of glutamates (Fig 5), which is associated with the enhanced glutamatergic synaptic transmission on the GABAergic neurons (Fig 6). These data indicate that astrocytic dysfunction in glutamate uptake and glutamatergic excitation in GABAergic neurons lead to GABAergic neuron excitotoxicity. Furthermore, an inhibition of glutamate receptors partially reverses extracellular acidosis-induced dysfunctions of GABAergic neurons (Figs 7 and 8). Thus, in addition to the role of intracellular protons in GABAergic neuron dysfunction, the impairment of cortical astrocytes leads to glutamate-mediated excitotoxicity to the GABAergic neurons during acidosis (Fig 9).

In terms of the mechanisms underlying acidosis-induced impairment in cerebral brains, previous studies indicate the roles of cellular metabolic changes, such as intracellular enzyme activity [6], proton accumulation [7,8] and acid-sensing ion channel [9–12]. These studies do not show what types of nerve cells, such as astrocytes or excitatory versus inhibitory neurons, are involved in these processes. It is found that GABAergic neurons are vulnerable to pathological factors [21,22] and acidosis leads to the brain dysfunction via impairing GABAergic neurons [15,24]. We focus on examining how GABAergic neurons are impaired by acidosis, especially the role of neural network. As neuron-glia interactions fulfill the brain functions [25–28], we focus on studying how the astrocytes interact with GABAergic neurons leading to acidosis-induced brain dysfunction. Our results in a neuron-specific manner and the astrocyte’s involvement reveal the novel mechanism for the impairment of GABAergic neurons under pathological conditions.

The astrocytes are believed to play a critical role in maintaining neuronal functions [38–41] and to be involved in some pathological conditions, e.g., ischemia and epilepsy [42–44]. Here, we find that the astrocytes are involved in the acidosis-induced dysfunctions of cortical GABAergic neurons, which adds new information into the astrocyte’s profiles in pathology. To prevent a role of the astrocytes in glutamate-related neurotoxicity, the future studies should be done to reveal the mechanism underlying the vulnerability of the astrocytes to pathological factors and the enhancement of astrocytic glutamate transporters.

The studies indicate that some pathological conditions, such as ischemia and acidosis, upregulate glutamatergic synapses [22,38] and Fig 6). The astrocytes and excitatory neurons interact each other through the excitatory synapses [34,45]. The impairment of the glutamate transporters on the astrocytes during acidosis is likely due to the activation of their glutamatergic synapses and the subsequent change of the astrocytic metabolism, a process of glutamate-related astrocytic toxicity. There may be many mediated-steps between extracellular acidosis and the final changes, such as intracellular signaling cascades [46] and acid-sensing ion channel [10]. Hypotheses remain to be tested by observing the changes in astrocytic metabolism and glutamate transporters during acidosis.

A future direction to address the role of cortical astrocytes in acidosis-induced neuron injuries is to use the mice whose astrocytes are genetically upregulated or downregulated, which will be supplement for our studies to quantify glutamate transporters on the astrocytes and to examine the effect of glutamate accumulation in their downstream. As the astrocytes play important role in the mammalian brain function, the mice with genetic manipulation in the astrocytes may not be well grown. In addition, the reagents that are currently applied to affect the central nervous system are not astrocyte-specific. Therefore, our studies will be strengthened once astrocyte-specific approaches are clearly developed.

Our study reveals that the extracellular acidification impairs the functions of GABAergic neurons including their spike production and synaptic outputs (Figs 1–4). The impairments may be responsible for psychological deficits in acidosis patients. The acidosis-induced impairment of spike encoding is due to the elevation of threshold potentials and the prolongation of refractory periods mediated by voltage-gated sodium channel (VGSC; Fig 2). Therefore, acidosis impairs the dynamics of VGSCs and GABA release at the cortical inhibitory neurons, which in turn causes the hyper-excitability in cerebral excitatory neurons and the deteriorations in cognitive behaviors, such as anxiety, convulsion and unconsciousness [2,3,5].

## Materials and Methods

### Brain slices and neurons

Entire procedures were approved by Institutional Animal Care and Use Committee in Anhui, China. The cortical slices (400 μm) were made from FVB-Tg(Gad GFP)4570Swn/J mice (Jackson Lab, Bar Harbor, ME 04609, USA) in postnatal day 22–25. These mice were anesthetized by inhaling isoflurane and decapitated by guillotine. The cortical slices in coronal direction were cut with Vibratome in the oxygenated (95% O_2_/5% CO_2_) artificial cerebrospinal fluid (ACSF) in concentrations (mM) of 124 NaCl, 3 KCl, 1.2 NaH_2_PO_4_, 26 NaHCO_3_, 0.5 CaCl_2_, 4 MgSO_4_, 20 dextrose, and 5 HEPES (pH 7.35 at 4°C). These slices were held in the oxygenized ACSF (124 NaCl, 3 KCl, 1.2 NaH_2_PO_4_, 26 NaHCO_3_, 2.4 CaCl_2_, 1.3 MgSO_4_, 10 dextrose and 5 HEPES with pH 7.35) at 25°C for 2 hours. Each slice was then transferred to a submersion chamber (Warner RC-26G) that was perfused by the oxygenated ACSF at 31°C for whole-cell recording [47,48]. Chemical reagents were purchased from Sigma.

GABAergic neurons for whole-cell recordings were selected in layer II~III of the sensory cortices based on GFP-labeled neurons under the DIC-fluorescent microscope (Nikon, FN-E600, Japan), in which an excitation wavelength was 488 nm. The neurons demonstrated fast spiking and less adaptation in spike amplitudes and frequencies, the typical properties for interneurons [49–52].

### Whole-cell recordings in the neurons and astrocytes

Cortical GABAergic neurons were recorded under the conditions of voltage-clamp for studying their responses to excitatory synaptic inputs and their inhibitory synaptic outputs as well as of current-clamp for analyzing their active intrinsic properties by an amplifier (AxoPatch 200B, Axon Instrument, Foster CA, USA). The electrical signals were inputted into Digidata 1440 and pClamp 10 (Axon Instrument Inc.) for the data acquisitions and analyses. The output bandwidth in this amplifier was 3 kHz. The pipette solution to record excitatory activities included (mM) 150 K-gluconate, 5 NaCl, 5 HEPES, 0.4 EGTA, 4 Mg-ATP, 0.5 Tris-GTP, and 5 phosphocreatine (pH 7.35; [53,54], and the pipette solution to record the inhibitory postsynaptic currents contained (mM) 135 K-gluconate, 20 KCl, 4 NaCl, 10 HEPES, 0.5 EGTA, 4 Mg-ATP, and 0.5 Tris–GTP (pH 7.35; [55]. The fresh pipette solution was filtered with centrifuge filters (0.1 μm). Its osmolarity was 295~305 mOsmol. The pipette resistance was 5~6 MΩ [54].

The functions of the cortical GABAergic neurons were assessed based on their intrinsic property, their responses to excitatory synaptic inputs and their GABAergic synaptic outputs [22,56]. Sequential action potentials at the GABAergic neurons were induced by injecting depolarization pulses whose intensities and durations were changed based on the aim of experiment under current-clamp. The inter-spike intervals induced by depolarization pulses (200 ms in duration) were used to assess the ability of GABAergic neurons to convert excitatory input signals into digital spikes [57]. Neuronal active intrinsic properties included spike threshold potentials (Vts) and absolute refractory period (ARP). Vts was the voltage to fire the spikes [52,58]. ARPs were measured by injecting paired-depolarization currents (3 ms) into the neurons after each spike (Fig 2). By changing inter-pulse intervals, we defined ARP as the duration from complete spike to its subsequent spike at 50% probability [59,60].

Excitatory synaptic transmissions on GABAergic neurons were examined by whole-cell voltage-clamp, in which spontaneous excitatory postsynaptic currents (sEPSC) were recorded without presynaptic stimulation in the presence of 10 μM bicuculline [22,61]. It was noteworthy that sEPSCs were blocked by washing 10 µM 6-Cyano-7-nitroquinoxaline-2,3-(1*H*,4*H*)-dione (CNQX) and 40 μM D-amino-5-phosphonovanolenic acid (D-AP5) into the slices before the end of the experiments, i.e., glutamatergic synapses. The synaptic outputs of GABAergic neurons to influence their target neurons was assessed by recording spontaneous inhibitory postsynaptic currents (sIPSC) on cortical pyramidal neurons under the voltage-clamp. 10 μM CNQX and 40 μM D-AP5 were added in the ACSF to block ionotropic glutamate receptor-channels. At the end of the experiments, 10 μM bicuculline was washed to the slices to test whether their synaptic responses were mediated by GABA_A_R [62,63]. The amplitudes of sEPSC and sIPSC represent the sensitivity and the density of the postsynaptic receptors. The frequencies of sEPSCs and sIPSCs represent the probability of transmitter release and the conversion of silent receptors into functional ones [62,64,65]. Therefore, these parameters can be used to analyze presynaptic and postsynaptic mechanisms, but the evoked postsynaptic currents cannot separate these mechanisms out. In addition, we did not applied TTX into the ACSF to record miniature postsynaptic currents since we had to record neuronal excitability. As the frequency of synaptic events was less than that of sequential spikes (Figs 1, 3 & 6) and the spontaneous spikes were never recorded on different types of the neurons in our study from cortical slices, sIPSCs and sEPSCs were not generated from spontaneous action potentials. Therefore, the synaptic events in our recordings would be thought as miniature postsynaptic currents. This point was supported by a single peak of the postsynaptic currents in our studies.

The function of the astrocytes was assessed by recording glutamate transporter current (GTC) under whole-cell voltage-clamp. As the processes of the astrocytes enclose the synapses formed by presynaptic terminals and postsynaptic spines [45], presynaptic released glutamates will activate astrocytic glutamate transporters for their re-uptake [66–68]. Astrocytic GTCs were evoked by stimulating presynaptic axons [35,69]. GTC amplitudes indicated the efficacy of glutamate clearance from synaptic clefts by the astrocytic glutamate transporters. It was noteworthy that 10 μM DL-threo-β-Benzyloxyaspartate (TBOA, a glutamate transporter antagonist from TOCRIS; [36,37] was applied to the cortical slices after the end of the experiments to make sure that GTCs were recorded. This result is showed in supplementary data (S1 Fig).

### In vitro acidosis

Acidosis was made by the two ways, extracellular acidification and intracellular acidification, to simulate pH changes in the internal environment for the brain cells. Extracellular acidosis was made by changing ACSF perfusion solutions into the brain slices from pH 7.35 to 6.75, in which all of the compositions between these two solutions were identical except for the difference in the pH values. This internal environment was close to the situation in clinical practices for acidosis. Under this condition, the changes of pH values would be executed precisely and all of the nerve cells including the astrocytes and neurons in the brain slices were affected by this acidification environment, i.e., the tissue acidosis. On the other hand, to simulate acidosis at an individual GABAergic neurons, we used the method of making a neuron to suffer from the acidification, i.e., intracellular acidosis, but not its neighbors. This cellular acidosis was induced by using a recording pipette to change intracellular pH values. The tip of pipettes contained the control pipette solution with pH 7.35, and then these pipettes were back-filled by the pipette solution with pH 6.75. The components in these two solutions were identical except for pH. With these pipettes for whole-cell recording, intracellular pH were balanced by the pipette pH in a few minutes. The neuronal functions recorded initially were thought to be the control. After a few minutes of the physical diffusion for pH balance between pipettes and neurons, the recorded neuronal signals were obtained under the condition of acidosis, i.e., a sequence from control to cellular acidosis. This approach is similar to the studies of infusing other reagents [7,47,70].

The data were analyzed if the recorded neurons had resting membrane potentials negatively more than -60 mV for the GABAergic neurons and -90 mV for the astrocytes. The criteria for the acceptance of each recording also included less than 5% changes in the resting membrane potential and input resistance from all nerve cells, as well as the spike magnitude in the neurons throughout each experiment. The input resistance was monitored by measuring cellular responses to hyperpolarization pulses at the same values as the depolarization that evoked action potentials. The presented values of spike frequencies were their averaged values at the maximal levels (plateau levels) of the input-output curves. The presented values in the amplitudes and inter-event intervals of sEPSCs and sIPSCs for the statistic comparisons before versus after acidosis or between extracellular acidosis and intracellular acidosis were their averaged values at 0.67 cumulative probability [55,71]. sEPSCs, sIPSCs, GTCs, inter-spike intervals, Vts and ARP were presented as mean±SE. The comparisons of these data before and after acidosis were done by paired t-test. The comparisons among the different groups were conducted by one-way ANOVA [72]. “*” and “#” indicate p<0.05. “**” and “##” indicate p<0.01.

## Supporting Information

S1 Fig Glutamate transporter currents are blocked by TBOA on cortical astrocytes. (DOC)
