# Supplementary material for: Acidosis-Induced Dysfunction of Cortical GABAergic Neurons through Astrocyte-Related Excitotoxicity
Source: PLoS One. 2015 Oct 16;10(10):e0140324. doi: 10.1371/journal.pone.0140324 (PMC4608795; doi:10.1371/journal.pone.0140324)
Supplement: S1 Fig — (DOC) [file pone.0140324.s001.doc]

**PLOS ONE**

**Acidosis-induced dysfunction of cortical GABAergic neurons through astrocyte-related excitotoxicity**

**
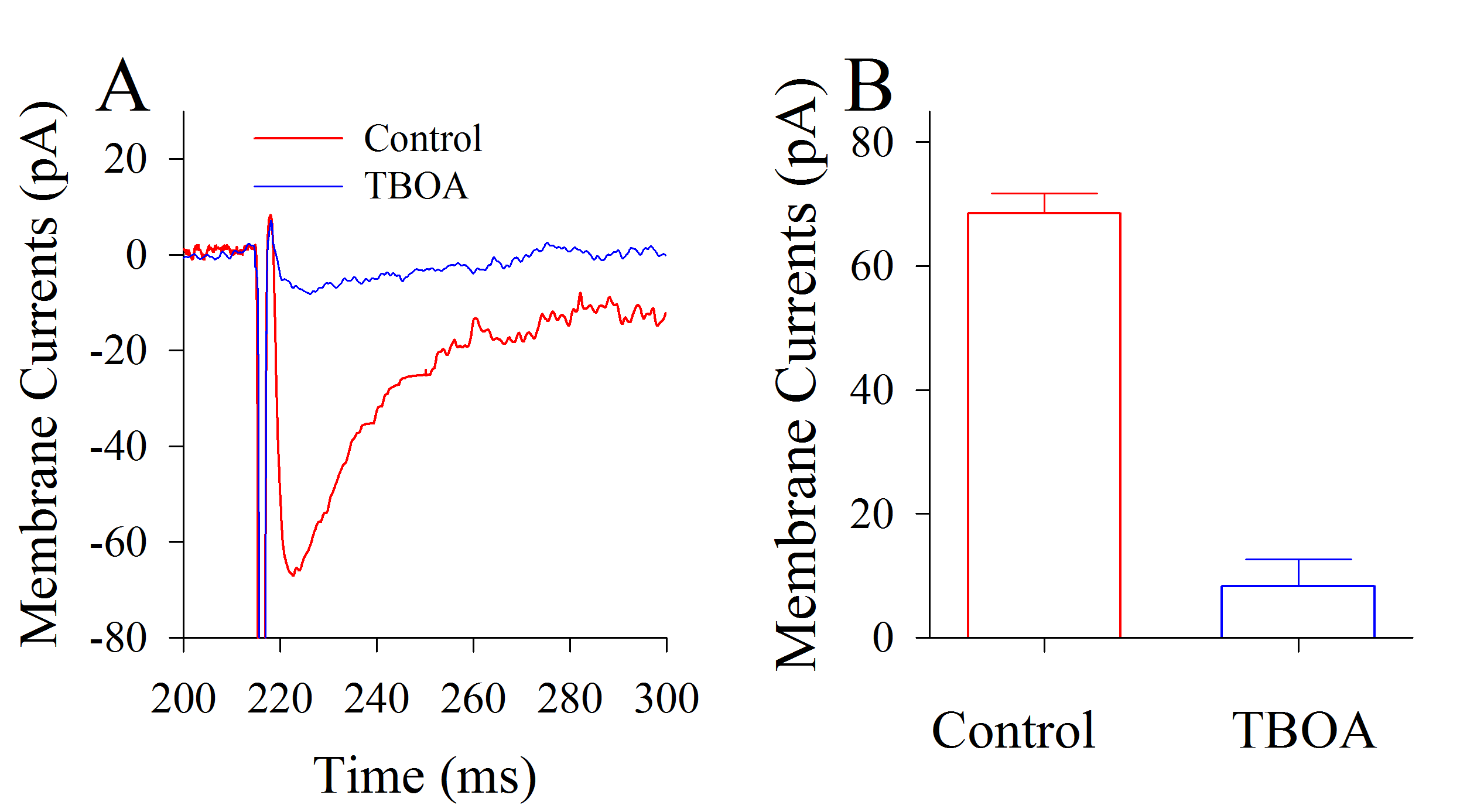
**

**S1 Fig** Glutamate transporter currents (GTC) are blocked by TBOA on cortical astrocytes. **A)** shows the superimposed waveforms of GTCs that are recorded on cortical astrocytes and evoked by stimulating presynaptic axons before (red trace) and after using TBOA (a glutamate transporter antagonist; 10 μM; blue trace). **B)** shows the averaged values of GTCs before (red bar) and after using TBOA.
